# Student responses to the introduction of case-based learning and practical activities into a theoretical obstetrics and gynaecology teaching programme

**DOI:** 10.1186/1472-6920-4-26

**Published:** 2004-11-29

**Authors:** Júlio Cesar Massonetto, Cláudio Marcellini, Paulo Sérgio Ribeiro Assis, Sérgio Floriano de Toledo

**Affiliations:** 1 Department of Maternal-infantile Health, Medical Sciences, Centro Universitário Lusíada, Rua Dr. Oswaldo Cruz 179, CEP-11045-101, Santos, Brazil

## Abstract

**Background:**

The fourth-year Obstetrics and Gynaecology course at our institution had previously been taught using theory classes alone. A new teaching model was introduced to provide a better link with professional practice. We wished to evaluate the impact of the introduction of case discussions and other practical activities upon students' perceptions of the learning process.

**Methods:**

Small-group discussions of cases and practical activities were introduced for the teaching of a fourth-year class in 2003 (Group II; 113 students). Comparisons were made with the fourth-year class of 2002 (Group I; 108 students), from before the new programme was introduced. Students were asked to rate their satisfaction with various elements of the teaching programme. Statistical differences in their ratings were analysed using the chi-square and Bonferroni tests.

**Results:**

Group II gave higher ratings to the clarity of theory classes and lecturers' teaching abilities (p < 0.05) and lecturers' punctuality (p < 0.001) than did Group I. Group II had greater belief that the knowledge assessment tests were useful (p < 0.001) and that their understanding of the subject was good (p < 0.001) than did Group I. Group II gave a higher overall rating to the course (p < 0.05) than did Group I. However, there was no difference in the groups' assessments of the use made of the timetabled hours available for the subject or lecturers' concern for students' learning.

**Conclusions:**

Students were very receptive to the new teaching model.

## Background

In Brazil, medical school courses last for six years and demand full-time study. In our school, the curriculum follows the traditional model, and it is divided into the basic cycle (first and second years), clinical cycle (third and fourth years) and pre-intern cycle (fifth and sixth years; full-time outpatient and hospital practice).

The practice of institutional self-evaluation, especially for educational institutions, has become part of the country's recent culture [1]. It has come about as a result of the democratic transition that Brazil went through from 1986 onwards and with the introduction of quality control principles in the past decade. In our school, we introduced an annual subject evaluation programme (SEP) in 2000, for the purposes of self-evaluation. Every year, in the middle of the second semester, all students in each year-group fill out a standard questionnaire that aims to assess each subject according to the following variables: teaching ability, teaching quality, lecturer's punctuality, student's improvement and commitment to each subject, test evaluation, stimulus given to discussion and clinical reasoning, guidance on practical activities, emphasis on the doctor-patient relationship, clinical correlation between the subject taught, and general impression. Each of these items is rated by the students as *very weak*, *weak*, *regular*, *good *or *very good*. The annual report consists of the evaluations on each subject, for all the above-mentioned variables, and is handed in to each lecturer in charge and the department representative [2].

The teaching of medicine centred on diseases and hospital care and, consequently, centred on the less prevalent disorders, has arisen as a consequence of the current curricular model. This has proven to be inadequate, inefficient and onerous for Brazil's health care sector. Students' participation in the health system is practically non-existent in the basic cycle of the current medical curriculum. In this light, over the last 15 years or so, there has been a series of movements among institutions, aiming towards changing the Brazilian medical school system [3].

In addition to the implementation of curricular directives of greater efficacy, other measures may stimulate medical students and better prepare them for professional practice. Such invigorating measures may include the linking of basic sciences with all the phases of the professional cycle, scientific initiation programmes that are accessible to all students, support programmes and academic guidance [4].

To adapt our medical sciences course to these new concepts within the Brazilian setting of health care and medical teaching, a committee for discussing and drawing up a new teaching system was set up. The process began in the second semester of 2000 and has gradually involved more and more of the lecturers, students, members of the board of directors and the school's sponsoring foundation.

Thus, in the new model that was proposed, problem-solving techniques would be the main teaching tool [5]. The model was also grounded in the basic principles of adult education: adults have a profound need for self-motivation [6] and must therefore take on an active role in the learning process. Adults are motivated much more to learn because of their own inner needs, such as their drive to succeed and satisfaction in learning in order to reach specific personal objectives, than because of outside factors [7].

Consequently, the content of the fourth-year programme was restructured for the 2003 academic year, with the aim of providing the new teaching tool of clinical case discussions alongside the learning of theory. Students' responses to these changes were assessed in comparison with the 2002 course. Our working hypothesis was that we would be promoting four positive actions: a) integration of theoretical and practical learning from the beginning of the students' contact with the speciality; b) greater consolidation of knowledge of the speciality; c) optimisation of the time that is made available for the speciality in the fourth year of the course; and d) preparation of the staff for the curricular model that would be implemented in the coming years.

## Methods

Each year-group of the medical course at Centro Universitário Lusíada (UNILUS) consists of 120 students. The Obstetrics and Gynaecology course is given in the fourth year (4 hours per week, giving a total of 120 hours) and the fifth and sixth years (a total of 925 hours for the pre-intern cycle). Up to and including 2002, the fourth-year course was taught by means of theory classes only, which aimed to cover all normal aspects of Obstetrics and Gynaecology and principal diseases encountered. Subsequently, during the pre-intern cycle, other relevant topics concerning disorders within the speciality were dealt with through visits to patients and seminars prepared by the students under staff supervision. In 2003, small-group discussions of cases and practical activities based on the normal aspects and diagnosis methods of the speciality were introduced for the fourth year. These activities replaced 50% of the theory content (the content relating to obstetrical and gynaecological pathology). The theory content taken from the fourth-year curriculum would be taught during the pre-intern cycle, together with the practical learning.

A large proportion of the staff was mobilised. Each week, during the four hours of teaching, students were divided into two groups. Sixty students attended two theory classes (one topic within Obstetrics and the other within Gynaecology), while the other sixty students were divided into six groups of ten students for discussions of clinical cases or practical activities. The two groups of sixty students alternated throughout the year. It should be stressed that the theory classes were taught by the same lecturers, using the same teaching material, during the two years of the present study.

The study began with a standardised SEP questionnaire that was administered to all fourth-year students in the second semester of the teaching year. This was done on the day of their assessment test, to ensure full attendance. The questionnaire bore the institution's official stamp and consisted of several questions, as described in the introduction, above. The questions used in the present study sought ratings for the use made of the timetabled hours available for the subject, lecturers' concern for students' learning, clarity of theory classes and lecturers' teaching abilities, lecturers' punctuality, quality of the assessment tests, students' learning of the subject and general evaluation of the subject. In the 2003 questionnaire, the variable *new methodology *– *activities in small groups *was introduced. Each of these items was rated by the students as *very weak *, *weak*, *regular*, *good *or *very good*. Group I consisted of 108 fourth-year students on the medical sciences course who answered the questionnaire in 2002 and group II consisted of 113 fourth-year students on the course in 2003.

The findings were tabulated using Microsoft ^® ^Excel 2002 for later evaluation. For analysis purposes, positive evaluations were considered to be the sum of the good and very good ratings, and negative evaluations the sum of the weak and very weak ratings. For statistical analysis, the variables were represented by absolute (n) and relative (%) frequency, and the difference between them was analysed using the chi-square test (χ^2^). The significance level adopted was 0.05 (α = 5%), and descriptive levels (p) that were less than this value were considered significant and marked by an asterisk (*). Significant values were also submitted to the Bonferroni test to ratify their statistical value.

## Results

Group I consisted of 66 female (61%) and 42 male students (39%), whose average age was 23.1 years, while group II consisted of 67 female (59%) and 46 male students (41%), whose average age was 23.6 years. There was no significant difference between their ages.

In group I, 95 students (88%) gave a positive rating for the use made of the timetabled hours available for the subject and 13 (12%) gave a regular rating for it, whereas 101 students in group II (89%) gave a positive rating and 12 (11%) a regular rating. There was no significant difference in this evaluation between the two groups (Table 1 – item 1). Eighty-five students (79%) in group I considered that the lecturers had great concern for students' learning, and 98 students (87%) in group II also believed this. Thus, there was no statistically significant difference between the groups (Table 1 – item 2).

**Table 1:** Distribution of course evaluation.

Question	Rating	Group I 2002 *n (%)*	Group II 2003 *n (%)*	χ^2^	*p*
1. Use made of timetabled hours available for the subject	1	95 (88%)	101 (89%)		
	2	13 (12%)	12 (11%)	0.1106079	> 0.05
	3	0	0		

2. Lecturers' concern for students'	1	85 (79%)	98 (87%)		
learning	2	14 (13%)	9 (8%)	2.4986106	> 0.05
	3	9 (8%)	6 (5%)		

3. Clarity of theory classes and lecturers' teaching abilities	1	72 (67%)	98 (87%)		
	2	31 (29%)	12 (11%)	12.765231	< 0.05 *
	3	5 (4%)	3 (2 %)		

4. Lecturers' punctuality	1	108 (100%)	81 (72%)		
	2	0	32 (28%)	32.620532	< 0.001 *
	3	0	0		

5. Quality of knowledge assessment tests	1	38 (35%)	73 (65%)		
	2	49 (45%)	34 (30%)	21.978341	< 0.001 *
	3	21 (20%)	6 (5 %)		

6. Students' learning of the subject	1	60 (56%)	97 (86%)		
	2	46 (42%)	13 (12%)	24.619225	< 0.001 *
	3	2 (2 %)	3 (2%)		

7. General evaluation of the subject	1	80 (74%)	100 (89%)		
	2	25 (23%)	10 (9%)	7.6007955	< 0.05 *
	3	3 (3 %)	3 (2%)		

Ratings: 1 – *Good and very good *2 – *Regular *3 – *Weak and very weak*

The clarity of theory classes and lecturers' teaching abilities received a positive evaluation from 72 students in group I (67%) and from 98 students (87%) in group II. This was a statistically significant difference (Table 1 – item 3).

Lecturers' punctuality received a positive evaluation from all 108 students in 2002, but only from 72% (81 students) in 2003, and this was statistically significant (Table 1 – item 4). Only 35% of group I (38 students) gave a positive rating for the quality of the knowledge assessment test, whereas 65% of group II (73 students) gave this a positive rating, which was a significant difference (Table 1 – item 5).

In 2002, 60 students (56%) rated their learning of the subject as good or very good, while in 2003, 97 students (86%) rated it as positive, which was a significant difference (Table 1 – item 6). Finally, the general evaluation of the subject was rated as good or very good by 80 students (74%) in 2002 and by 100 students (89%) in 2003, which was a statistically significant difference (Table 1 – item 7).

The new methodology adopted in 2003 for the Obstetrics and Gynaecology course was considered to be good or very good by 89% of the students, regular by 8% and weak or very weak by 3%.

## Discussion

The traditional curriculum model was developed with reference to the Flexner report of 1910 [8]. In this, medical education was considered to be a process of initiation in a science. The teachers' role was to establish what students must learn, to transmit information that was considered relevant, and to evaluate students' capacities to retain and reproduce the information presented. Theory would be dealt with before practice, with the aim of preparing students for the use of theory during students' internship and subsequent professional lives. In this model, medical practice is detached from scientific practice, thereby promoting fragmentation of knowledge and neglect of the psychosocial and cultural aspects of medical activities [9]. This teaching approach has been criticised for the excessive value given to content and for its low efficacy, which brings about the subsequent need for re-qualification. We believe that this "banking concept of education" that Freire [10] refers to is conclusively condemned to history.

On the other hand, the teaching concept of meaningful learning calls for linkage between the roles of universities, health care administrators and social services. It suggests that there should be co-operation in the selection of content, production of knowledge and development of professional competence. In meaningful learning, the teacher is no longer the main source of information, but the facilitator of the teaching-learning process. The teacher's aim is to stimulate the learner to take on an active, critical and reflective attitude in the knowledge building process. The content dealt with must have the potential to be meaningful (functionality and relevance for professional practice), giving value to matters that are pertinent and correlatable with students' cognitive structure. However, the absorption by students of knowledge of the so-called basic subjects in this context presents a great challenge [11].

The curricular directives for medical courses (Report 583/01, of August 7, 2001) from the Brazilian National Education Council (part of the Ministry of Education) give guidance on the changes to be made to the teaching model for courses. They indicate that courses must involve students in practical activities from the outset and promote active integration between health care service users and professionals from the beginning of their instruction, using methodology which reinforces students' active participation in knowledge-building, thereby bridging the gap between academic medical learning and the social needs of Brazilian health care. It is evident that the new curricular directives have used the concepts and logic of problem-based learning as their reference point. They have been based on various American and European curricula that, over the past decade, have been giving emphasis to free time for self-study instead of traditional lectures [12-14]. Thus, more than half of the medical schools in the United States are at present undergoing a process of curricular reform [15], as are a large proportion of the medical schools in the United Kingdom [16,17].

**Figure 1:**
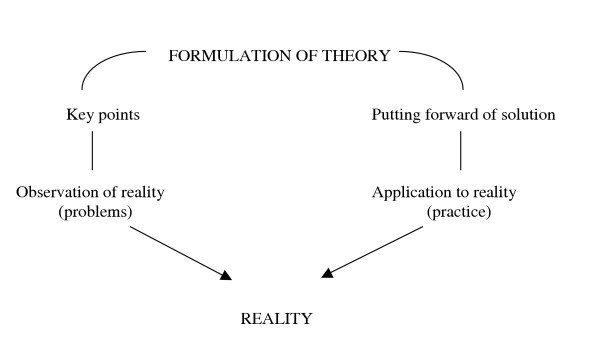
Maguerez's Arch.

In the "problematization" methodology based on Maguerez's Arch, as presented by Bordenave [18], five phases develop from reality: observation, key points, formulation of theory, putting forward of solution and application to reality (Figure 1). This is an alternative methodology that is appropriate to higher education. It differs significantly from problem-based learning in some points that are summarised in Table 2 (adapted from Berbel, 1998 [19]).

**Table 2:** Main differences between "*problematization*" *and problem-based learning*.

	"Problematization"	Problem-based learning
Observation of reality	Problems constructed by the lecturers of subjects in which this methodology is used (subject option)	Construction of problems by the lecturers, with complete vertical and horizontal integration (institutional option)
Key points	Not defined	Defined in the curriculum
Formulation of theory	Investigation-guided study	Investigation-guided study
Putting forward of solution	Done after study	Done by students before study, on the basis of previous knowledge
Application to reality (practice)	Results must intervene in reality as much as possible	Intervention in the social environment is considered to be fundamental

In problem-based learning, the cognitive objectives are all previously established, while in "problematization", total control over the resultant knowledge does not exist. The essence of problem-based learning is that the problems define objective concepts to be learned and non-objective concepts that can be excluded from the learning because they are not relevant to the study in question [16].

Although it may be difficult and scientifically dangerous to compare results from conventional curricula (lecture-based learning) and models like problem-based learning or "problematization" [20-23], this was not our intention. Our only objective was to evaluate a teaching tool that is already well known and make a contribution towards discussions on curricular reform.

The present study does not prove that the "modernised" curriculum is better than the previous one, but it emphasises that the strengths of the "new" curriculum are worthy of more exploration. In our opinion, the perception that a qualitative improvement in students' learning has taken place during the course is the first step towards a more substantial and effective change in the teaching-learning process. In the present study, the intention was to transform a totally theoretical course into a more stimulating and efficient course. In this, concepts acquired during classes would be applied clinically to real cases obtained by the students themselves in the wards. A recent study at Manchester University [16] has shown that changing a conventional course into a new integrated course, using problem-based learning throughout, has significantly improved recently graduated students' perceptions of their preparedness for entering the professional market.

There was no significant difference in students' evaluations of the use made of the time available for the subject between the two groups, because there was already a positive assessment among the 2002 group (Table 1 – item 1). Likewise, students gave positive evaluations regarding their perception of lecturers' concern for their learning. Although there was no significant difference between the groups in relation to this question, there was a mild tendency towards increased positive evaluation among the 2003 group (Table 1 – item 2).

An improvement in the assessment of the course can be seen from item 3 of Table 1 onwards. From 2002 to 2003, there was a significant increase in the positive rating given to clarity and teaching abilities in the classes taught. At first, this seemed odd to us, considering that the teaching material used and the staff who taught the theory classes were identical for the two groups. We concluded that the insertion of clinical cases and practical classes into the traditionally theoretical course was the decisive factor in students' perception that the 2003 lessons had improved. Although the fact that the questionnaire was administered at the time of the final assessment test may have had an influence on the data, the questionnaire was administered on the same occasion for each of the two year-groups.

The decrease in the rating of lecturers' punctuality can be easily explained by the fact that the theory classes were always predictably held in the same place in 2002 (group I), while group II used various locations that were specially booked for them. On some occasions in 2003, unexpected events occurred at the beginning of the activities (Table 1 – item 4).

Assessment tests for Obstetrics and Gynaecology are traditionally considered to be difficult. There was a perception in our school that they did not reflect the overall knowledge of the subject that is required. The tests consist of forty to fifty multiple-choice questions (each with five alternatives presented) and five essay-type questions. The former perception can be seen among the 2002 year-group in item 5 of Table 1, alongside the significant improvement among the 2003 group. This indicates to us that the 2003 year-group studied with greater satisfaction and interest, stimulated by the new process, and that this group consequently made the interpretation that there was greater coherence in the preparation of tests. Nonetheless, the tests did not undergo any substantial change from 2002 to 2003. Despite this improvement in the rating, we are still far from achieving the desired positive evaluation rate for the quality of our tests, and the present study shows us that the tests need to be improved.

One of the most important objectives in a change in the teaching system is to obtain greater course efficiency and increased student learning. Items 6 and 7 of Table 1 show us that, at least with regard to student perceptions, this aim has been achieved. Our assessment is that the change in the teaching system was very stimulating for the development of students' study routines. The holistic concept of modern education directs us towards integrating knowledge, understanding and practice for learners. In this, learning is taken to be an ongoing part of life and not just a preparation for it [24]. In keeping with this view, the medical curriculum needs to drum into students the ethos of self-evaluation [7].

Students responded well to the new method, as shown by the positive rating of 89% given by the 2003 year-group. This provides us with the basis for further advances in this subject in the years to come. It gives the staff the confidence to institute significant changes in the curricular reform that has been under discussion for three years.

Although the staff's level of satisfaction was not an objective of our study, initial observation of this indicates great commitment to the course and, probably, better performance. However, it will only be through future longitudinal studies that we will know whether there has really been greater consolidation of knowledge and course efficiency.

## Conclusions

Students were very receptive to the new teaching model in this study. An active role in their learning process seems to be more pleasant and productive than usual method. Thus, active learning methodology should be stimulated on the medical courses throughout the world.

## Competing interests

The author(s) declare that they have no competing interests.

## Authors' contributions

All authors participated in the study design and application. JCM was the study coordinator. All authors read and approved the final manuscript.

## Pre-publication history

The pre-publication history for this paper can be accessed here:



## References

[B1] Troncon LEA, Figueiredo JFC, Rodrigues MLV, Peres LC, Cianflone ARL, Picinato CE, Colares MFA (1999). Implantação de um programa de avaliação terminal do desempenho dos graduandos para estimar a eficácia do currículo na Faculdade de Medicina de Ribeirão Preto. Rev Ass Med Brasil.

[B2] Massonetto JC, Dinato MC, Moretti PH (2002). Programa de Avaliação Disciplinar (PAD): instrumento para aperfeiçoamento pedagógico / Subject Evaluation Programme (SEP): tool for pedagogic improvement. Rev Bras Educ Med.

[B3] Feuerwerker LCM (2002). *Mudanças na Educação Médica: os casos de Londrina e Marília*. São Paulo: Hucitec; Londrina: Rede Unida; Rio de Janeiro: Associação Brasileira de Educação Médica.

[B4] Schanaider A (2002). Integração das ciências básicas e áreas profissionais no ensino de graduação em Medicina / Cooperation between basic sciences and professional areas in undergraduate medical education. Rev Bras Educ Med.

[B5] Foster M, Dornan T (2003). Self-directed, integrated clinical learning through a sign-up system. Med Educ.

[B6] Illesca PM, Navarro HN (2002). Aprendizaje centrado en el estudiante en la formación de los profesionales de la salud. Rev Chil Cs Med Biol.

[B7] Burge SM (2003). Undergraduate medical curricula: are students being trained to meet future service needs?. Clin Med.

[B8] Flexner A (1910). *Medical Education in the United States and Canada*.

[B9] Aguiar AC (2001). Implementando as novas diretrizes curriculares para a educação médica: o que nos ensina o caso de Harvard? / Implementing the new curricular guidelines for medical education: what does the Harvard case teach us?. Interface comum saude educ.

[B10] Freire P (1972). *Pedagogy of the Oppressed*.

[B11] Sweeney G (1999). The challenge for basic science education in problem-based medical curricula. Clin Invest Med.

[B12] Regehr G, Martin J, Hutchinson C, Murnaghan J, Cuisamano M, Reznick R (1995). The effect of tutors' content expertise on student learning, group process and participant satisfaction in a problem-based learning curriculum. Teaching Learning Med.

[B13] O'Neill PA (2000). The role of basic sciences in a problem-based learning clinical curriculum. Med Educ.

[B14] Distlehorst LH, Robbs RS (1998). A comparison of problem-based learning and standard curriculum students: three years of retrospective data. Teaching Learning Med.

[B15] Hollander H, Loeser H, Irby D (2002). An anticipatory quality improvement process for curricular reform. Acad Med.

[B16] Jones A, McArdle PJ, O'Neill PA (2002). Perceptions of how well graduates are prepared for the role of pre-registration house officer: a comparison of outcomes from a traditional and an integrated PBL curriculum. Med Educ.

[B17] O' Neill PA (1995). Problem-based learning at medical school and has been introduced successfully in Manchester. BMJ.

[B18] Maguerez C, Bordenave JD (1985). *Alguns fatores pedagógicos, capacitação pedagógica para instrutor, supervisor da área de Saúde*.

[B19] Berbel NAN (1998). A problematização e a aprendizagem baseada em problemas: diferentes termos ou diferentes caminhos? Problematization and problem-based learning: different words or different ways?. Interface comum saude educ.

[B20] Foley RP, Polson AL, Vance JM (1999). Review of the literature on problem based learning in clinical settings. Teaching Learning Med.

[B21] Albanese M (2001). Problem-based learning: why curricula are likely to show little effect on knowledge and clinical skills. Med Educ.

[B22] Norman GR, Schmidt HG (2001). Effectiveness of problem-based learning curricula: theory, practice and paper darts. Med Educ.

[B23] Komatsu RS (2002). Sobre a dificuldade da realização de estudos avaliando o desempenho de estudantes que desenvolvem diferentes currículos: comparando o incomparável? / About the difficulty of realization studies of evaluation of students that working different curricula. Rev Bras Educ Med.

[B24] Margetson DB (2000). Depth of understanding and excellence of practice: the question of wholeness and problem-based learning. J Eval Clin Pract.

